# Correction: Passive and active DNA methylation and the interplay with genetic variation in gene regulation

**DOI:** 10.7554/eLife.01045

**Published:** 2013-06-14

**Authors:** Maria Gutierrez-Arcelus, Tuuli Lappalainen, Stephen B Montgomery, Alfonso Buil, Halit Ongen, Alisa Yurovsky, Julien Bryois, Thomas Giger, Luciana Romano, Alexandra Planchon, Emilie Falconnet, Deborah Bielser, Maryline Gagnebin, Ismael Padioleau, Christelle Borel, Audrey Letourneau, Periklis Makrythanasis, Michel Guipponi, Corinne Gehrig, Stylianos E Antonarakis, Emmanouil T Dermitzakis

Gutierrez-Arcelus M, Lappalainen T, Montgomery SB, Buil A, Ongen H, Yurovsky A, Bryois J, Giger T, Romano L, Planchon A, Falconnet E, Bielser D, Gagnebin M, Padioleau I, Borel C, Letourneau A, Makrythanasis P, Guipponi M, Gehrig C, Antonarakis SE, Dermitzakis ET. 2013. Passive and active DNA methylation and the interplay with genetic variation in gene regulation. *eLife* **2**:e00523. doi: http://dx.doi.org/10.7554/eLife.00523. Published 4 June 2013

The affiliations for Stephen B Montgomery were incorrect, and there were errors in affiliations 1 and 2. The correct author affiliations are:

Maria Gutierrez-Arcelus^1,2,3^, Tuuli Lappalainen^1,2,3^, Stephen B Montgomery^1,4^, Alfonso Buil^1,2,3^, Halit Ongen^1,2,3^, Alisa Yurovsky^1,2,3^, Julien Bryois^1,2,3^, Thomas Giger^1,2^, Luciana Romano^1,2,3^, Alexandra Planchon^1,2,3^, Emilie Falconnet^1,2^, Deborah Bielser^1,2^, Maryline Gagnebin^1,2^, Ismael Padioleau^1,2,3^, Christelle Borel^1,2^, Audrey Letourneau^1,2^, Periklis Makrythanasis^1,2^, Michel Guipponi^1,2^, Corinne Gehrig^1,2^, Stylianos E Antonarakis^1,2^, Emmanouil T Dermitzakis^1,2,3^

^1^Department of Genetic Medicine and Development, University of Geneva Medical School, Geneva, Switzerland; ^2^Institute of Genetics and Genomics in Geneva, Geneva, Switzerland; ^3^Swiss Institute of Bioinformatics, Geneva, Switzerland; ^4^Departments of Pathology and Genetics, Stanford University, Stanford, United States

The article has been corrected accordingly.

